# Recent Views on Cerebro-Spinal Meningitis

**Published:** 1908-10-10

**Authors:** 


### RECENT VIEWS ON CEREBRO-SPINAL MENINGITIS.

Sufficient time has now elapsed since the recent widespread epidemic of cerebro-spinal meningitis to enable some definite conclusions to be drawn. At the recent meeting of the British Medical Association at Sheffield workers from many of the great centres affected by the epidemic of 1905-6 gave their experiences. The results of their prolonged researches add much to our knowledge of the subject, and at the same time lead us to anticipate a satisfactory and reliable method of treatment.

The main points brought forward may be summarised under the headings of (1) Diagnosis; (2) Relation to the endemic type of posterior basic meningitis of children; (3) Paths of infection; and (4) Treatment.

### Diagnosis.

The only reliable diagnosis must depend on a bacteriological examination of the cerebro-spinal fluid obtained by means of lumbar puncture.

Despite the fact that some observers have found examples of polymorphism and variations in staining characters of the organism, it is agreed that the epidemic type of disease is always due to the meningo-coccus of Weichselbaum. This is a diplococcus presenting definite morphological and cultural characteristics and readily found in the cerebro-spinal fluid, both within the cells and free in the fluid.

Of course, many other varieties of organisms produce meningitis, and even anthrax and typhoid bacilli have been found; and in two cases reported by McDonald a leptothrix was isolated and cultivated. But where organisms such as this last are found in epidemic districts it must be remembered that the meningo-coccus is but feebly resistant, and so might readily die out or be overgrown by some saprophytic organism after it has exerted its pathogenic effects.

In ordinary cases there is present in the fluid a number of polymorphonuclear leucocytes which may be sufficiently numerous to form definite pus. This purulent exudation into the meningeal space is occasionally so thick that it blocks the exploring syringe and prevents any fluid escaping, and so complicates the diagnosis.

Another point in diagnosis is the agglutination phenomenon. Rankin and Houston conclude from a large number of experiments in the Belfast epidemic that the blood serum of affected patients agglutinates the meningo-cocci, and they consider this test as reliable as Widal's reaction for typhoid fever. But from the fact that the serum takes several days to develop this agglutinative faculty, it can never have the same diagnostic import as the bacteriological examination of the lumbar puncture fluid.

### Relation to Endemic Post Basic Meningitis.

The diplococcus of Still found in the endemic type of posterior basic meningitis of children is in most respects identical with the meningo-coccus, and though Rankin and Houston, deducing from their agglutination experiments have decided that the organisms are different, others regard them as merely variations of the same type, and, indeed, the two diseases are alike clinically, pathologically, and microscopically.

### Paths of Infection.

The fact that the meningo-coccus is not infrequently found in the nose and cranial sinuses has led to the suggestion of a direct spread of infection from the nose to the meninges, but it is more probable that the nasal cavities are infected from the meninges. Meningitis over the inferior parts of the frontal lobes is not common, as it would be in direct infection from the nose, and, further, experimentally produced meningitis in monkeys has resulted in the finding of the organisms in the nasal mucosa. In some cases there is a general infection, and in many the cocci have been isolated from the blood and viscera. The path of infection is as yet unsettled.

The symptoms of this disease are toxic, from the absorption of bacterial products; and mechanical, from the pressure of inflammatory exudate around the cord and brain. It is important to remember that in cases which become chronic the exudation may organise around the foramen of Majendie and interfere with the free circulation of cerebro-spinal fluid, and so produce a condition of increasing hydrocephalus. Hence the object of treatment is to overcome the organisms as early in the disease as possible.

### Serum Treatment.

Several curative sera have been produced by various workers by immunising horses against various strains of meningo-cocci obtained from human cases, the process of immunisation lasting four or five months. Of these two deserve careful attention—namely, Flexner and Jolling's serum prepared at the Rockefeller Institute in New York, and Kolle and Wassermann's serum. With the latter good results have been obtained on the Continent, and with the former universally good results in America, Belfast, and other places.

### Flexner's Serum.

Emmett Holt, basing his statistics on nearly 500 cases treated with Flexner's serum, reports a marked reduction in the death-rate, and he anticipates as favourable a result from this serum as is seen with diphtheria antitoxin. The earlier the serum therapy is begun the better the prognosis. All are agreed that the serum must be brought into direct contact and in a concentrated form with the focus of disease, and subcutaneous or even intravenous injections are of little use. Large doses, 30 c.c., must be injected into the cerebro-spinal canal, and it is advisable to allow some of the cerebrospinal fluid to escape previously. If the purulent exudate in the meningeal space is so thick as to prevent the flow of fluid it is advisable to inject a few syringefuls of normal saline and then let it flow away.

The serum is definitely bacteriolytic, though it may be slightly antitoxic also. In favourable cases there is a marked effect on the inflammatory process; the pyrexia diminishes, sometimes 4° or 5°, the leucocytosis is less marked, the nervous symptoms abate and consciousness may return, and in twenty-four hours the meningo-cocci are considerably reduced in numbers and vitality, and after two injections they can no longer be cultivated. If no improvement occurs the dose is to be repeated in twelve hours, and then daily until the temperature falls.

### Kolle's Serum.

Kolle employs simultaneous intra-spinal and intra-venous injections, so as to destroy any organisms which may have found their way into the blood stream. Emmett Holt, in accentuating the importance of early treatment, advises immediate serum therapy if the fluid from the diagnosing lumbar puncture be turbid, and without waiting for bacteriological confirmation.

And, lastly, owing to the presence of certain antibodies in the blood of patients and convalescents, Mackenzie and Martin have tried in some cases injections of sera obtained from such cases, with results sufficiently hopeful to stimulate further work on these lines.

